# miR-144-3p通过靶向调控IRS1抑制肺腺癌细胞的侵袭和转移

**DOI:** 10.3779/j.issn.1009-3419.2021.104.05

**Published:** 2021-05-20

**Authors:** 俊 白, 雅琼 胡, 新璐 陈, 琳 陈, 丽萍 张, 崇高 尹, 洪利 李

**Affiliations:** 1 261053 潍坊, 潍坊医学院病理学教研室 Department of Pathology, Weifang Medical University, Weifang 261053, China; 2 261053 潍坊, 潍坊医学院护理学院 Colloge of Nursing, Weifang Medical University, Weifang 261053, China; 3 261053 潍坊, 潍坊医学院医学研究实验中心 Medical Research Center, Weifang Medical University, Weifang 261053, China

**Keywords:** 肺肿瘤, miR-144-3p, 侵袭转移, 胰岛素受体底物1, Lung neoplasms, MiR-144-3p, Invasion and metastasis, Insulin receptor substrate 1

## Abstract

**背景与目的:**

MicroRNAs（miRNAs）是短的非编码RNA，是能够调控基因表达、影响细胞过程、促进疾病发展的重要分子。在许多疾病中可以观察到miRNA表达的变化，包括肝炎、心血管疾病和癌症。本研究旨在探讨miR-144-3p靶向调控胰岛素受体底物1（insulin receptor substrate 1, IRS1）对肺腺癌细胞侵袭和转移的影响。

**方法:**

通过生物信息学数据库查询miR-144-3p在肺腺癌患者中的表达情况；利用mirTarPathway对miRNA进行KEGG通路富集分析；定量逆转录聚合酶链式反应（quantitative reverse transcription polymerase chain reaction, qRT-PCR）检测miR-144-3p在肺腺癌细胞系中的表达情况与质粒转染效率；Transwell实验检测不同组细胞侵袭迁移能力的变化。生物信息学确定miR-144-3p的关键基因（Hub基因）；双荧光素酶靶标实验检测miR-144和IRS1的相互结合情况；Western blot实验检测IRS1在不同细胞系的表达及过表达miR-144后IRS1的表达情况。

**结果:**

miR-144-3p在肺腺癌组织中表达降低，qRT-PCR结果提示miR-144-3p在肺腺癌细胞A549中的表达显著降低（*P* < 0.05）且过表达质粒转染成功（*P* < 0.05）；过表达miR-144后，细胞的迁移、侵袭和增殖能力下降（*P* < 0.05）；IRS1在肺腺癌组织中表达上调；生存分析表明高表达IRS1的肺腺癌患者预后不良（*P* < 0.05）；双荧光素酶实验结果显示，miR-144可特异识别IRS1的3’-UTR抑制报告酶的表达（*P* < 0.05）；Western blot结果提示IRS1在A549细胞中表达升高（*P* < 0.05），过表达miR-144后，IRS1蛋白表达水平下降（*P* < 0.05）；Transwell实验证明，miR-144-3p可通过靶向调控IRS1抑制肺腺癌细胞的侵袭和转移（*P* < 0.05）。

**结论:**

miR-144-3p通过靶向调控IRS1抑制A549细胞的侵袭和迁移能力，进而在肿瘤中发挥抑癌作用。

## Abstract (en)

**Background and objective:**

MicroRNAs (miRNAs) are short non-coding RNAs that regulate gene expression, influence cellular processes, and promote disease development. Variations in miRNA expression have been observed in many diseases, including hepatitis, cardiovascular disease, and cancer. The aim of this study is to investigate the effect of miR-144-3p on the invasion and metastasis of lung adenocarcinoma by targeting recombinant insulin receptor substrate 1 (IRS1).

**Methods:**

The expression of miR-144-3p in patients with lung adenocarcinoma was queried through bioinformatics database. MirTarPathway was used to analyze the KEGG enrichment pathway of miRNA. The expression and plasmid transfection efficiency of miR-144-3p in lung adenocarcinoma cell lines were detected by quantitative reverse transcription polymerase chain reaction (qRT-PCR). Transwell assay was used to detect the changes of cell invasion and migration ability in different groups. Bioinformatics determined the key genes (Hub genes) of miR-144-3p; Double luciferase target assay was used to detect the mutual binding of miR-144 and IRS1. Western blot assay was used to detect the expression of IRS1 in different cell lines and the expression of after overexpression of miR-144.

**Results:**

The expression of miR-144-3p in lung adenocarcinoma tissues was decreased, qRT-PCR results indicated that the expression of miR-144-3p in lung adenocarcinoma cell A549 was significantly decreased (*P* < 0.05), and the overexpressed plasmid was successfully transfected (*P* < 0.05). Overexpression of miR-144 decreased the ability of cell migration and invasion (*P* < 0.05). The expression of IRS1 was up-regulated in lung adenocarcinoma tissues. Survival analysis showed that patients with lung adenocarcinoma with high IRS1 expression had a poor prognosis (*P* < 0.05). Double luciferase assay results showed that miR-144 could specifically identify 3'-UTR of IRS1 and inhibit reporter enzyme expression (*P* < 0.05). Western blot indicated that the expression of IRS1 was increased in A549 cells (*P* < 0.05). After overexpression of miR-144, the expression level of IRS1 protein was decreased (*P* < 0.05). Transwell experiment proved that miR-144-3p could inhibit invasion and metastasis of lung adenocarcinoma cells by targeting IRS1 (*P* < 0.05).

**Conclusion:**

MiR-144-3p inhibits the invasion and migration of A549 cells through targeted regulation of IRS1, thus playing an anticancer role in tumors.

在全人类中，肺癌仍然是最常见的死亡原因^[1]^。其中非小细胞肺癌（non-small cell lung cancer, NSCLC）约占肺癌的85%，肺腺癌（lung adenocarcinoma, LUAD）占NSCLC的60%^[2]^。目前虽然新技术和新疗法在早期诊断中取得了进步，但LUAD患者的5年总生存率仍然很低^[3]^。因此，深入分析LUAD的转移机制具有重要意义。

MicroRNAs（miRNAs）通过互补作用负向调控基因表达，与靶基因mRNA的3'-非翻译区（3'-UTR）特异性结合^[4]^。miRNA与细胞上皮间质转化等多种细胞和生理过程相关，且其异常表达与多种人类肿瘤的进展和转移有关^[5]^。有研究^[6]^显示miR-144-3p可抑制胃癌细胞进展，其表达水平与肿瘤大小、淋巴结转移、肿瘤原发灶-淋巴结-转移（tumor-node-metastasis, TNM）分期和侵袭转移相关。胰岛素受体底物1（insulin receptor substrate 1, IRS1）是一种潜在致癌基因，参与胶质母细胞瘤、骨肉瘤的相关调控进展^[7, 8]^。有研究^[9]^表明，miR-144通过靶向IRS1在喉鳞癌中起到抑癌作用。但是，关于miR-144-3p靶向IRS1的调控LUAD机制仍有待研究。因此本研究将探讨miR-144-3p是否可以通过靶向调控IRS1影响LUAD的侵袭和转移，以期为LUAD治疗提供新方向。

## 1 材料与方法

### 1.1 材料与仪器

细胞株：人正常肺上皮细胞BEAS-2B和肺腺癌NCI-H1299、A549细胞均购自ATCC，并通过STR细胞鉴定。miR-144-3p引物由上海生工生物工程有限公司设计合成。双荧光素酶报告基因载体、质粒等均由吉凯基因构建合成。Lipofectamine2000购于Invitrogen公司，8 µm孔径Transwell小室购自BD Biosciences。IRS1抗体为兔抗IRS1抗体（ab40777），β-actin抗体为兔抗β-actin抗体（ab8227），抗体均购自Abcam公司。

### 1.2 生物信息学数据库

GEO数据库：从基因表达谱GEO数据库（https://www.ncbi.nlm.nih.gov）中下载肺腺癌患者资料。GSE51853数据集为5例正常组织与76例肺腺癌组织miRNA表达谱。Kaplan-Meier Plotter（Kmplot）网站（https://kmplot.com/analysis/）能够评估21种癌症类型中54, 000基因对生存率的影响。Starbase数据库（http://starbase.sysu.edu.cn/index.php）提供了miRNA和各种RNA分子的相互作用信息，并在此基础上构建了ceRNA网络。使用在线预测网站miRWalk（http://mirwalk.umm.uni-heidelberg.de/）、TargetScan（http://www.targetscan.org/）和miRDB（http://mirdb.org/miRDB/）预测miRNA的靶基因。使用STRING在线网站（https://string-db.org/）构建蛋白互作网络图，导入Cytoscape3.7.1删除独立节点，对蛋白互作网络图进行分析，它主要是通过节点、边缘、度和网络结构来测量网络，因此它可以帮助识别关键基因和关键蛋白质群落。

### 1.3 细胞培养与分组

常温复苏正常肺上皮细胞BEAS-2B，肺腺癌细胞NCI-H1299和A549于37 ℃、5%CO_2_的恒温培养箱中培养，待其生长至对数期，铺于六孔板中进行转染，质粒由上海吉凯生物有限公司构建。将细胞分组：①con组：将过表达miR-144对照质粒转入A549细胞；②miR-144组：转入miR-144过表达质粒；③A549/miR-144+NC组：同时转入miR-144过表达质粒和IRS1过表达对照质粒；④A549/miR-144+IRS1组：同时转入miR-144过表达质粒和IRS1过表达质粒。

### 1.4 定量逆转录聚合酶链式反应（quantitative reverse transcription polymerase chain reaction, qRT-PCR）

实验RNA提取及逆转录过程参考本课题先前已发表文献^[4]^。反应条件为95 ℃ 5 s、63 ℃ 30 s、72 ℃ 30 s进行35个循环。以U6作为内参。miR-144-3p的上游引物为3'-GCGCGCGTACAGTATAGATGA-5'，下游引物为5'-AGTGCAGGGTCCGAGGTATT-3'，茎环结构为5'-GTCGTATCCAGTGCAGGGTCCGAGGTATTCGCACTGGATACGACAGTACA-3'。

### 1.5 Transwell迁移侵袭实验

Transwell实验分析A549细胞的迁移和侵袭能力。将150 µL转染的A549细胞（4×10^4^个）悬液接种在含1%胎牛血清的培养基于上室；同时，将含有10%胎牛血清的培养基（500 µL）加入下室。与迁移实验不同的是，在侵袭实验中使用的Transwell小室涂有基质胶。24 h后，甲醇固定后用Giemsa染色，在显微镜下观察计数，所有实验均重复3次。

### 1.6 CCK8细胞增殖实验

收集各组细胞，将细胞悬液接种到96孔板中，每孔约100 μL、2×10^3^个细胞，培养24 h后每孔加入含10%CCK8的培养基10 μL，培养1 h后，测定吸光度值*A*450 nm，分别检测转染24 h、48 h、72 h、96 h后的细胞吸光度值*A*450 nm。独立重复实验3次。

### 1.7 Western blot实验

转染后的A549细胞，用RIPA裂解液提取总蛋白，进行分离胶浓度为12%的SDS-PAGE电泳，转膜，封闭，一抗4 ℃孵育过夜，在1:5, 000稀释的HRP二抗中孵育1 h，ECL曝光。一抗稀释度：IRS1为1:1, 000、β-actin为1:1, 000。

### 1.8 双荧光素酶实验

293T细胞购自ATCC，细胞培养方式按照ATCC建议。构建pGL3-IRS1-3'-UTR-MUT和pGL3-IRS1-3'-UTR-WT质粒，将293T细胞培养于24孔板中，100 ng的pGL3-IRS1-3'-UTR-MUT和pGL3-IRS1-3'-UTR-WT使用Lipofectamine2000（Invitrogen, 12566014）和miRNA对照及过表达载体分别共转染293T细胞。培养48 h，用萤火虫荧光值和海肾荧光值的比值计算荧光素酶报告基因的活性。

### 1.9 统计学分析

采用SPSS 17.0进行统计学分析，所有实验数据均用均数±标准差（Mean±SD），两组计量资料采用独立样本*t*检验，*P* < 0.05为差异具有统计学意义。

## 2 结果

### 2.1 miR-144-3p在肺腺癌组织中表达降低以及KEGG（Kyoto Encyclopedia of Genes and Genomes）通路分析

通过对GEO2R分析并获取数据集GSE51853，筛选条件为logFC < -1（*P* < 0.05）结果发现miR-144-3p在GSE51853数据集中的表达下调且*P*值最小；显示miR-144-3p在LUAD组织中表达降低，且差异显著（图 1A、图 1B）。此外Starbase数据库结果显示，miR-144-3p在LUAD患者组织中的表达较正常肺腺组织降低（图 1C）。我们用mirTarPathway对miR-144-3p进行KEGG通路分析，以明确miR-144-3p在各种通路的富集情况，从而对其发挥调节作用的机制进行初步预测。结果显示，miR-144-3p在p53信号通路以及TGF-beta信号通路产生富集，这些通路在癌症中都发挥着至关重要的作用^[10]^，因此我们可以推测miR-144-3p也许通过参与相关通路的调控从而影响肺腺癌的进展（图 1D）。

**图 1:**
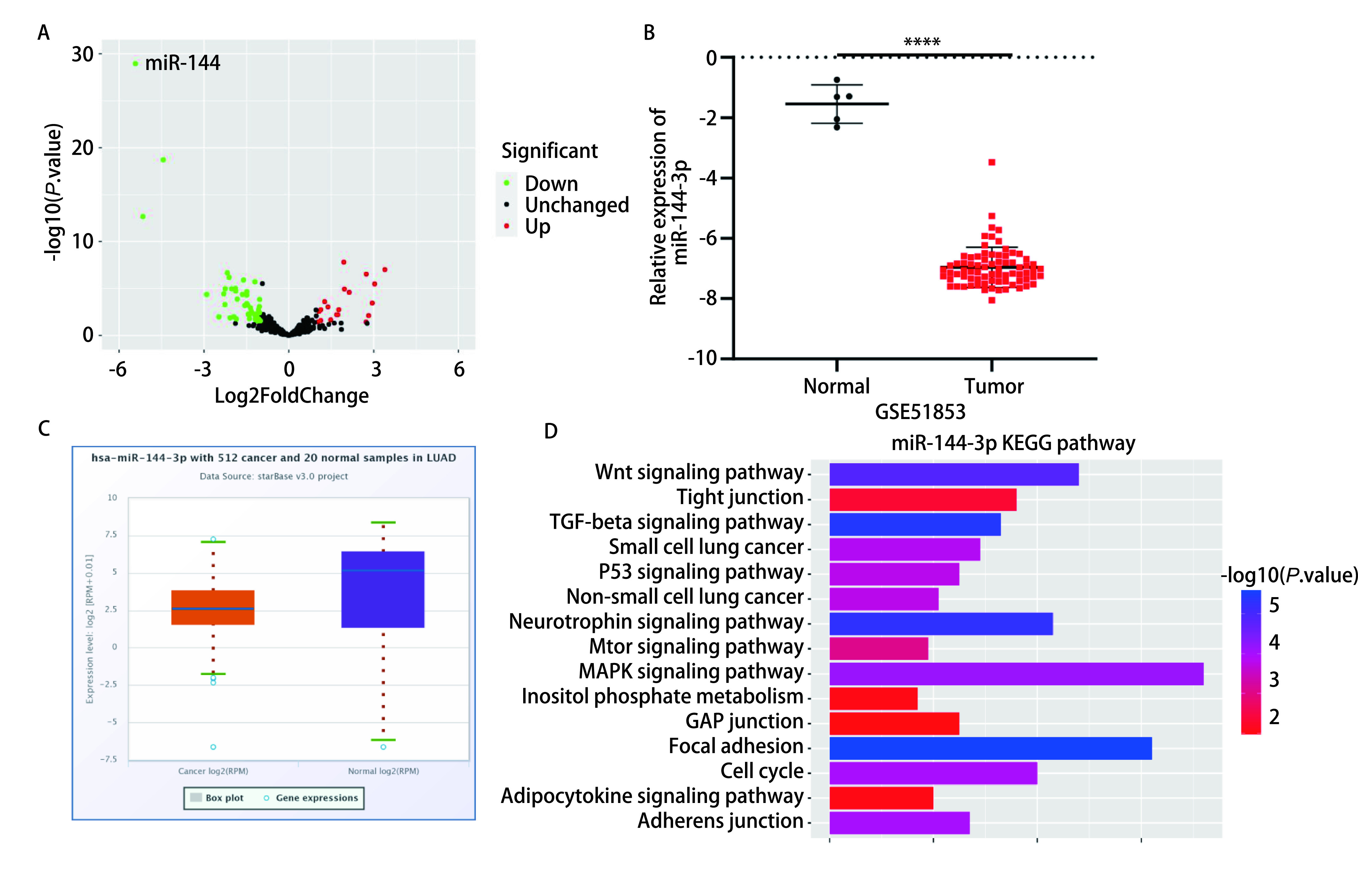
miR-144-3p在LUAD患者组织中的表达量和miR-144-3p的KEGG通路分析。A：火山图显示miR-144-3p在数据集GSE51853的表达差异；B：miR-144-3p在正常和肺腺癌组织中的表达情况；C：Starbase查询miR-144-3p的表达情况；D：KEGG通路分析miR-144-3p的富集情况。 Expression of miR-144-3p in lung adenocarcinoma tissue and its KEGG pathway analysis. A: Volcano plot of GSE51853 showing miRNA expression in lung adenocarcinoma tissues and normal tissues; B: The expression of miR-144-3p in normal tissues and cancer tissues; C: Starbase queries the expression of miR-144-3p; D: KEGG pathway analysis the enrichment of miR-144-3p. LUAD: lung adenocarcinoma; TGF: transforming growth factor; KEGG: Kyoto Encyclopedia of Genes and Genomes. ^*^^*^^*^^*^P < 0.0001.

### 2.2 miR-144-3p在肺腺癌细胞中低表达

qRT-PCR检测结果显示：BEAS-2B、H1299、A549细胞株中miR-144-3p相对表达量分别为1.00±0.00、0.75±0.05、0.54±0.03，因此选取A549细胞进行后续研究（*P* < 0.05，图 2A）。qRT-PCR检测转染miR-144过表达质粒A549/miR-144后miR-144-3p的表达情况，结果显示与A549/con组（1.00±0.00）相比，A549/miR-144组中的miR-144-3p（2.70±0.06）表达水平显著增高（*P* < 0.05，图 2B），提示转染成功。

**图 2:**
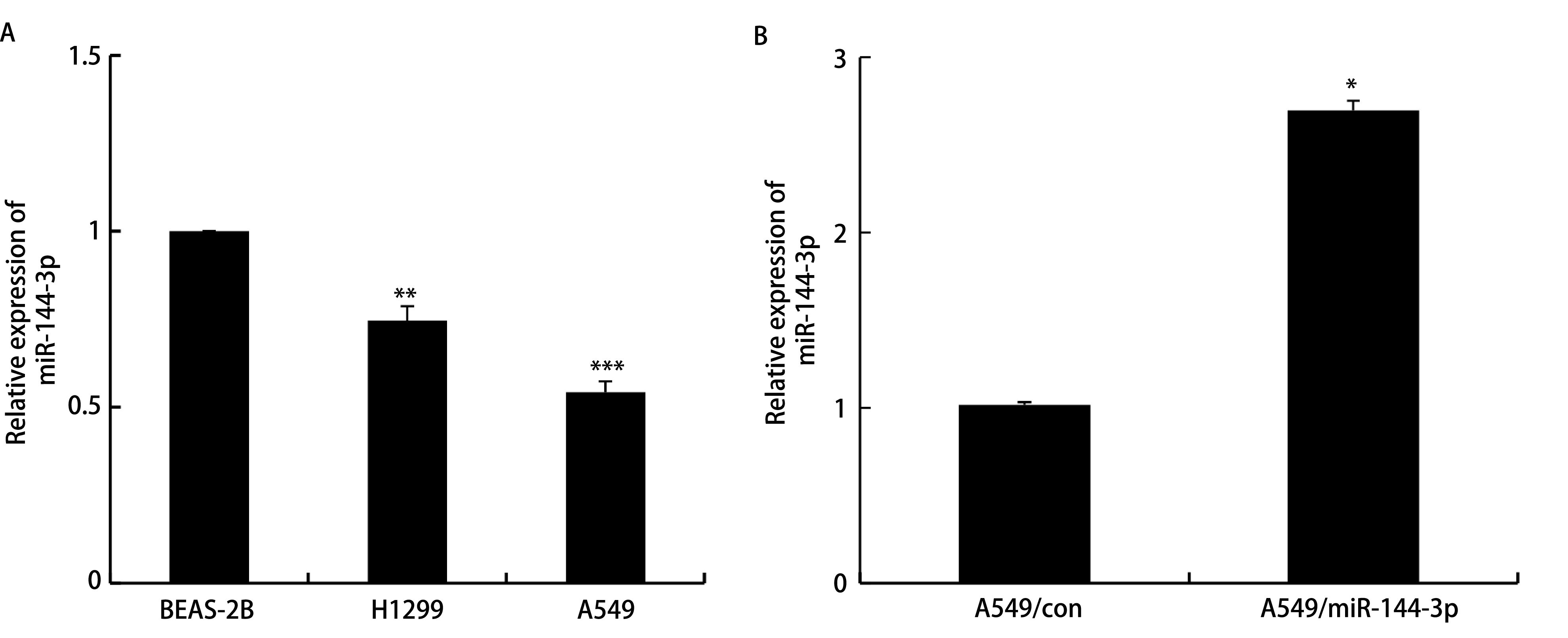
miR-144-3p在各组细胞中的表达量。A：qRT-PCR验证miR-144-3p在不同细胞系的表达情况（^*^^*^P < 0.01，^*^^*^^*^P < 0.001）；B：qRT-PCR验证miR-144-3p的转染效率。 Expression of miR-144-3p in different cells. A: qRT-PCR verifies the expression of miR-144-3p in different cell lines (^*^^*^P < 0.01, ^*^^*^^*^P < 0.001); B: qRT-PCR verifies the transfection efficiency of miR-144-3p (^*^P < 0.05). qRT-PCR: quantitative reverse transcription polymerase chain reaction.

### 2.3 过表达miR-144抑制肺腺癌细胞的侵袭转移和增殖能力

Transwell迁移实验显示，A549/miR-144组细胞穿过下室的细胞数（169.33±4.04）比A549/con组细胞数（240.00±21.17）明显减少（*P* < 0.05)；侵袭实验结果显示，A549/miR-144组细胞穿过基底膜的细胞数（155.33 ±5.03）比A549/con组细胞数（205.00±5.00）明显减少（*P* < 0.05）（图 3A、图 3B），结果证明过表达miR-144可以抑制肺腺癌细胞的侵袭转移。通过CCK8细胞增殖实验检测各组细胞的增殖能力，结果显示，与对照组在72 h（0.65±0.05）、96 h（0.96±0.02）的增殖能力相比，过表达miR-144组在72 h（0.43±0.07）、96 h（0.67±0.06）的增殖能力明显降低。

**图 3:**
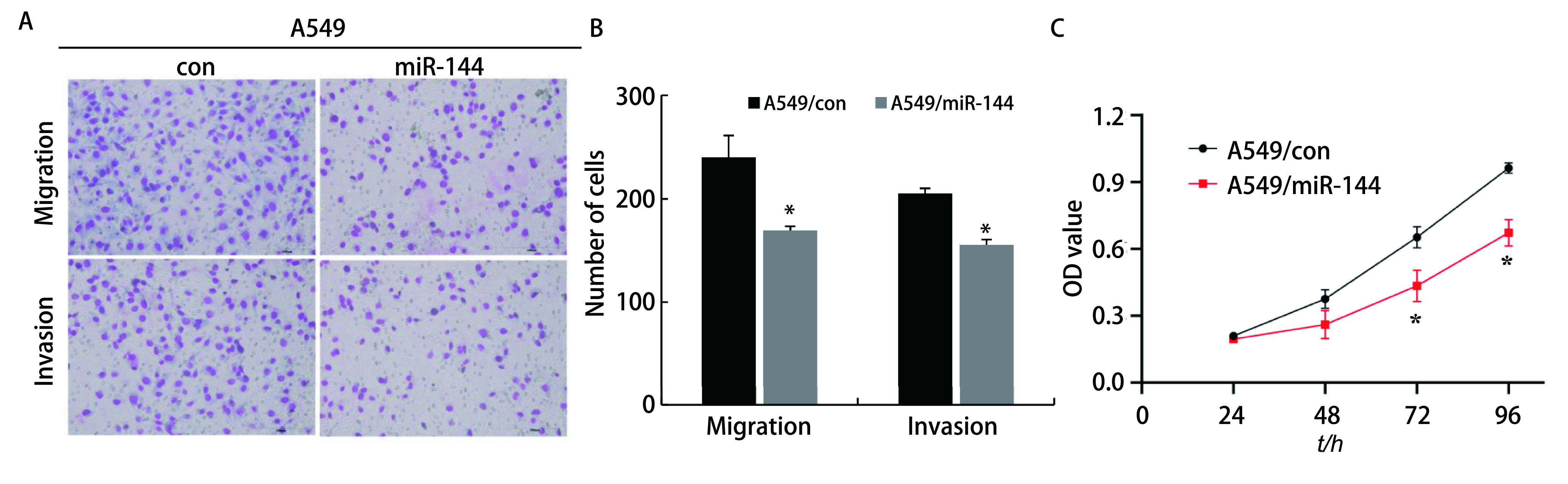
过表达miR-144抑制A549细胞的迁移侵袭和增殖能力。A：Transwell实验验证各组细胞的迁移和侵袭能力；B: Transwell实验检测各组细胞的统计学分析（^*^P < 0.05）；C：CCK8细胞增殖实验检测各组细胞的增殖能力（^*^P < 0.05）。 Overexpression of miR-144 inhibits the migration and invasion and proliferation ability of of each group. A: Transwell assay verified the ability of cell migration and invasion in each group; B: Transwell assay was used to detect the statistical analysis of cells in each group (^*^P < 0.05); C: CCK8 cell proliferation assay was used to detect the proliferation ability of each group (^*^P < 0.05). OD: optical density.

### 2.4 *IRS1*为miR-144-3p的Hub基因

利用预测软件miRDB、miRWalk、TargetScan预测miR-144-3p的靶基因，交集得到122个靶基因（图 4A）。我们使用STRING分析miR-144-3p靶基因间的蛋白相互作用关系，并构建蛋白互作网络，根据基因间的相互作用强度及置信度运用Cytoscape绘制蛋白互作网络并进行筛选。结果显示，degree值的前十位基因为（*TJP1*、*ZEB2*、*FMR1*、*FN1*、*ZEB1*、*GRM5*、*CREB1*、*PBRM1*、*IRS1*、*SMARCA4*）（图 4B、图 4C），其中颜色由蓝色到橘色，表示degree值由小到大，并且degree值越高，节点的大小越大，结合分数越高，边的宽度越宽。GEDs在线数据库查询IRS1在癌症中的表达情况，与正常组织相比，IRS1在LUAD中表达升高（图 4D），且高表达水平的IRS1与患者不良预后相关（*P*=0.018，图 4E）。因此我们选定IRS1进行下一步研究。

**图 4:**
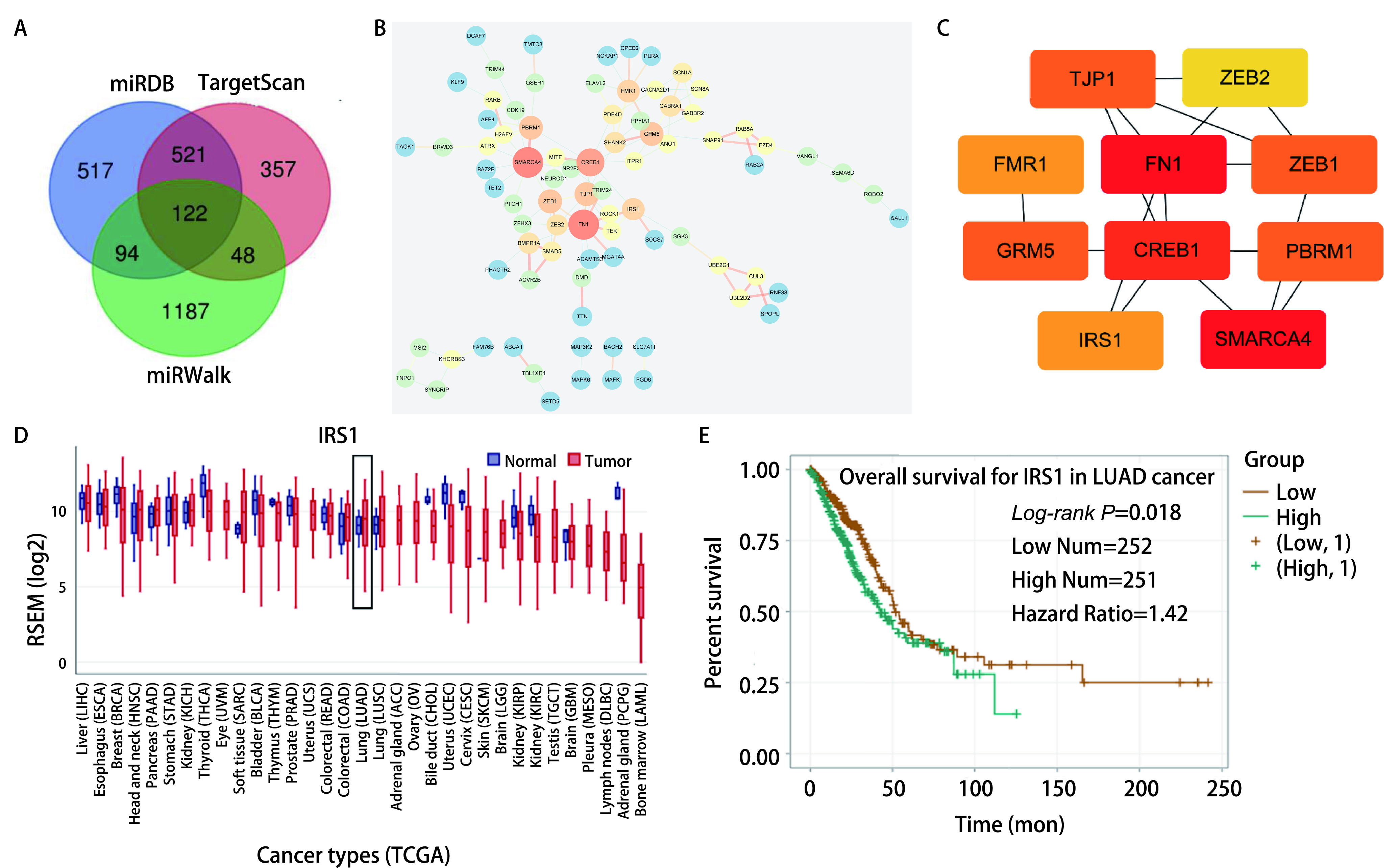
miR-144-3p的Hub基因筛选。A：基因预测维恩图；B：PPI互作网络；C：Cytoscape检测靶向基因的枢纽基因；D：IRS1在正常组织和肺腺癌组织中的表达；E：IRS1的生存曲线分析。 Hub gene screening of miR-144-3p. A: The venn plot of predicted genes; B: PPI interaction network; C: The Hub genes of targeted genes made by Cytoscape; D: IRS1 expression in normal lung adenocarcinoma tissues and lung adenocarcinoma tissues; E: Survival analysis of of IRS1 (*P*=0.018). PPI: protein protein interaction.

### 2.5 miR-144-3p通过靶向调控IRS1抑制LUAD细胞的侵袭转移能力

通过双荧光素酶实验检测，将miR-144质粒与pGL3-IRS1-3'-UTR-WT报告载体共转染后，荧光素酶活性降低（*P* < 0.05）；而将miR-144质粒与pGL3-IRS1-3'-UTR-MUT报告载体共转染后，荧光素酶活性无明显变化（图 5A）。结果表明IRS1 mRNA的3'-UTR是miR-144的直接结合位点。Western blot实验显示，IRS1在A549细胞（1.64±0.10）中的表达明显高于BEAS-2B（1.00±0.04）（*P* < 0.05，图 5B），在A549/miR-144组细胞中的IRS1蛋白表达（0.62±0.03）明显比A549/con组细胞的（1.00±0.12）低（*P* < 0.05，图 5C）。Transwell迁移实验结果显示miR-144+IRS1组穿过基底膜的细胞数（313.33±10.41）比miR-144+NC组细胞数明显增多（133.33±7.64），侵袭实验结果显示miR-144+IRS1组穿过基底膜的细胞数（224.67±9.61）比miR-144+NC组细胞数明显增多（113.67±7.09）且差异显著（*P* < 0.05，图 5D）。结果表明，IRS1 mRNA的3'-UTR是miR-144的直接结合位点，且miR-144可以通过靶向调控IRS1抑制LUAD细胞的迁移和侵袭能力。

**图 5:**
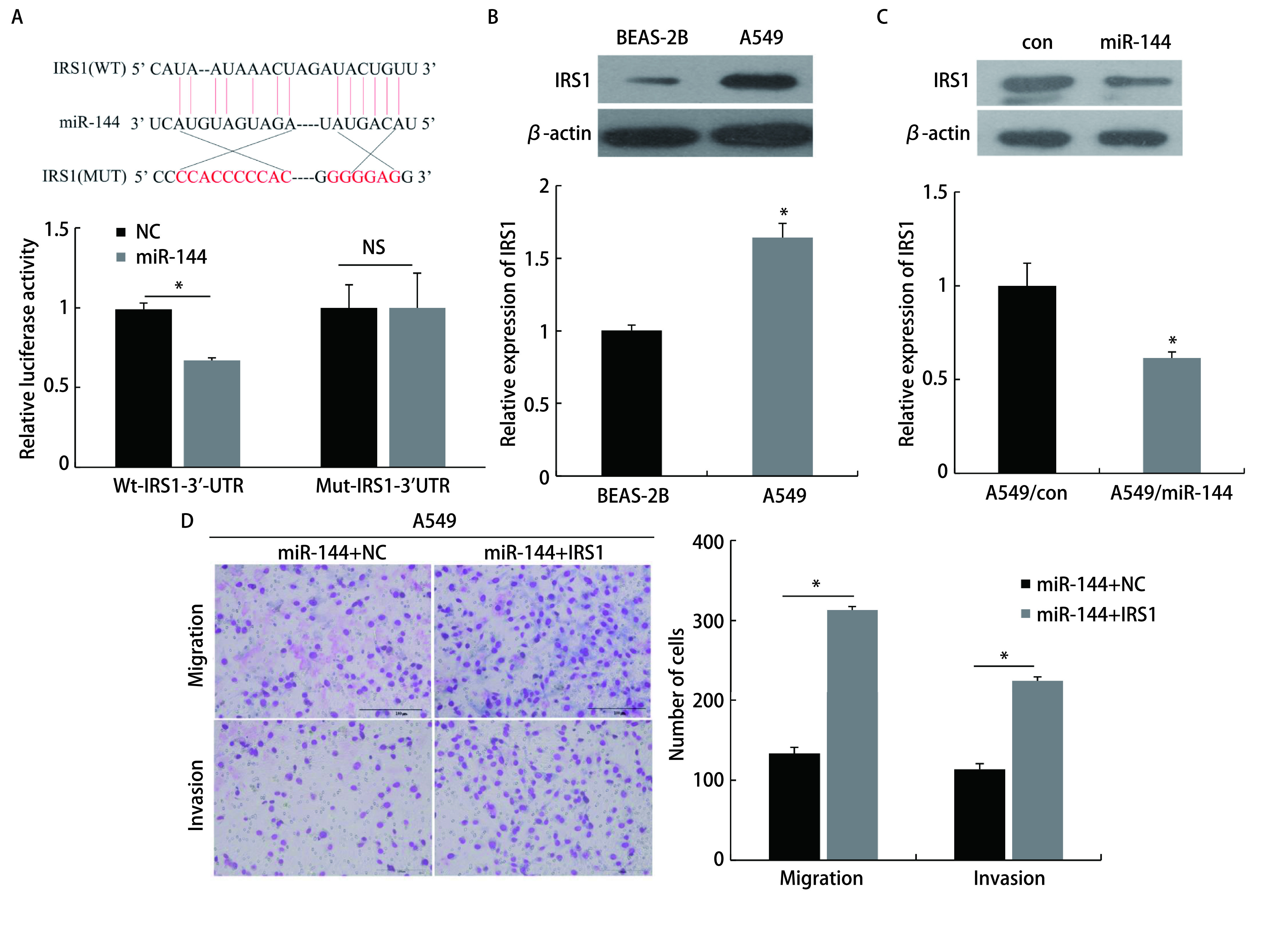
miR-144靶向调控IRS1抑制肺腺癌细胞的迁移和侵袭能力。A：荧光素酶实验检测不同组细胞的荧光素酶活性；B：Western blot验证IRS1的表达；C：Western blot检测过表达miR-144后IRS1的表达情况；D：Transwell实验检测不同组细胞的迁移和侵袭能力。 miR-144 inhibits the invasion and migration ability of lung adenocarcinoma cancer by targeting the regulation of IRS1. A: Luciferase activity of cells in different group was detected by luciferase experiment; B: Western blot to verify IRS1 expression; C: Western blot to detect the expression of IRS1 after overexpression of miR-144; D: Transwell experiment detects the migration and invasion ability of different groups of cells. NS: no significance.

## 3 讨论

肿瘤的侵袭和转移在肺癌最初诊断时经常见到，是肺癌相关死亡的主要原因^[11]^。目前已经报道了许多关于miRNA对肺癌的调节，例如，miR-193阻止NSCLC的侵袭和迁移^[12]^。miR-454作为一种预后因素，对肺癌细胞的增殖和转移做出了贡献^[13]^。关于人类癌症的研究，miR-144的异常下调已在多种人类恶性肿瘤中被证实，包括胆管癌、结直肠癌、膀胱癌和甲状腺癌^[14]^。

*IRS1*是一种潜在的致癌基因，与多种恶性肿瘤有关^[15]^。在功能上，IRS1不仅诱导转化和肿瘤发生，而且是多种致癌途径的枢纽。研究表明，IRS1可能作为癌基因参与癌细胞的生长、增殖、迁移、侵袭和分化^[16]^，并且在胰腺癌^[17]^、结直肠癌^[18]^、胶质母细胞瘤^[19]^中高表达。IRS1异常表达与预后差、复发率高和恶性肿瘤的生存率相关，我们当前的目的是揭示IRS1在LUAD中发挥作用的机制^[20]^。

有证据^[21]^表明miR-144还可以通过靶向ZFX抑制肝癌细胞的增殖和转移。miR-144通过靶向TIGAR抑制细胞增殖，促进肺癌细胞凋亡和自噬^[22]^。LUAD复发和转移的潜在机制尚不清楚。本次实验研究了microRNA-144-3p（miR-144-3p）在LUAD发生和进展中的作用。通过由GEO数据库中的GSE51853数据集，筛选得到了与LUAD相关的miR-144-3p，生物信息学数据库显示miR-144-3p在LUAD中表达降低，KEGG通路富集分析发现miR-144-3p与癌症通路相关。qRT-PCR检测miR-144-3p在肺腺癌细胞中的表达情况。在体外，miR-144-3p上调降低细胞存活率和迁移率，反之亦然。有研究^[23]^表明，XIST通过抑制miR-144调控IRS1的表达和PI3K/AKT信号通路促进喉鳞癌的进展。本研究通过PPI和CytoHubba识别候选基因，最终选取IRS1作为研究对象。另外GEDS数据库以及Kmplot数据库分析了IRS1在正常与癌症组织中的表达，生存分析以及靶向结合情况。在机制上，miR-144-3p能够负调控相关转移蛋白IRS1。我们的结果支持miR-144-3p通过靶向IRS1参与LUAD的进展，因而它有可能成为LUAD潜在的生物标志物和治疗靶点，后续我们的实验将继续研究miR-144-3p在相关信号通路中的作用。

综上所述，miR-144-3p与靶基因IRS1很有可能参与LUAD的发生发展过程，这对完善miR-144-3p与LUAD的关系提供了参考资料。对开拓miR-144-3p在LUAD中的研究提供了新的方向。
